# Complete genome sequence of *Streptococcus oralis* subsp. *tigurinus* J22, a model strain for antagonistic interaction against *Streptococcus mutans*

**DOI:** 10.1128/mra.00677-25

**Published:** 2025-10-24

**Authors:** Hee-Young Jung, Jina-Na Cai, Dongyeop Kim

**Affiliations:** 1 Department of Preventive Dentistry, School of Dentistry & Institute of Oral Bioscience, Jeonbuk National University 26714, Jeonju, Republic of Korea; 2 Department of Oral Biology, School of Stomatology, Binzhou Medical University 698075 , Yantai, People's Republic of China; Nanchang University, Nanchang, Jiangxi, China

**Keywords:** oral commensal, hydrogen peroxide, antagonism, biofilm

## Abstract

*Streptococcus oralis* subsp. *tigurinus* strain J22, a potent hydrogen peroxide-producing oral commensal. Given the antagonistic action against a cariogenic pathogen, this strain can be used as a model bacterium to characterize microbial antagonistic interactions. *S. oralis* J22 has a genome sequence of 1,967,320 bp along with 41.1% GC content.

## ANNOUNCEMENT

**Fig 1:**
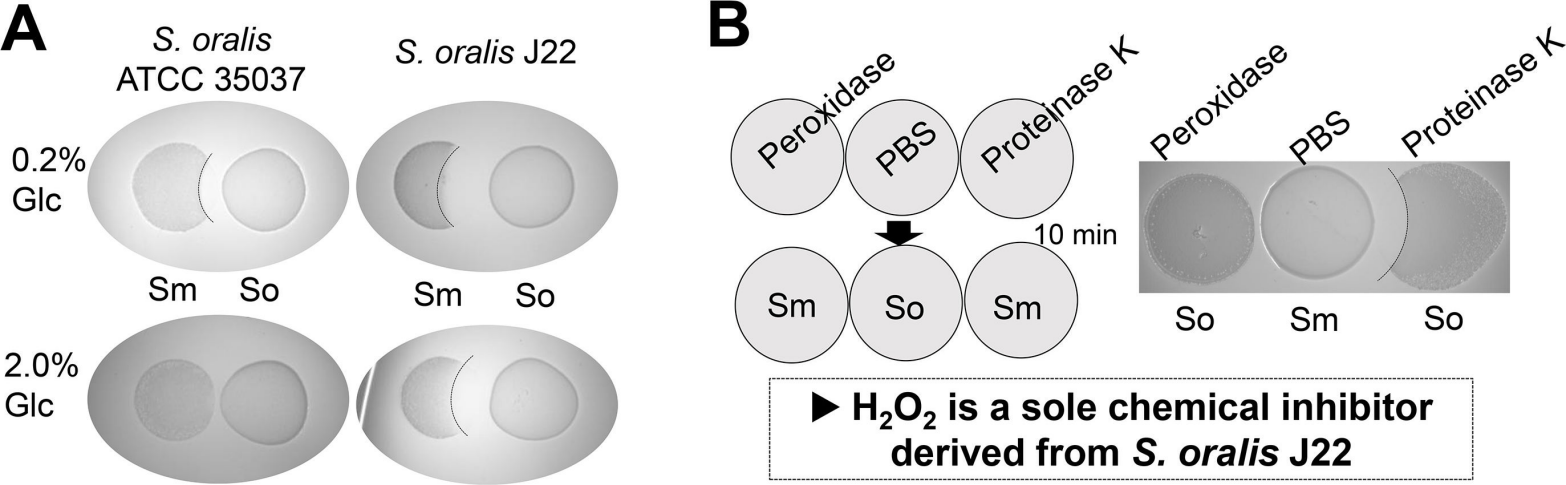
Representative antagonistic activity of *S. oralis* J22 against *S. mutans*. (**A**) *S. oralis* J22 consistently inhibited *S. mutans* growth regardless of sugar concentration in the agar medium. In contrast, *S. oralis* ATCC 35037 exhibited sugar-dependent inhibition, with markedly reduced activity under high-glucose conditions (at 2% glucose compared to 0.2% glucose). (**B**) To test whether H_2_O_2_ is a sole chemical weapon generated by *S. oralis* J22, either peroxidase or protease was applied for 10 min prior to spotting of *S. mutans* in the proximity of *S. oralis*. Interestingly, the peroxidase completely diminished the inhibitory zone mediated by *S. oralis*, whereas the protease did not show any drastic changes in the growth inhibition when compared to the medium without treatments.

*Streptococcus oralis* subsp. *tigurinus* strain J22 is a potent hydrogen peroxide (H_2_O_2_)-producing oral commensal. It has been used as a model bacterium to study bacterial antagonistic interactions against oral pathogens and to evaluate the inhibitory capacity of oral isolates in suppressing cariogenic biofilm formation (1–4). Its antagonistic activity is mediated by H_2_O_2_ production rather than proteinaceous compounds (e.g., bacteriocins) (Fig. 1).

Compared to other oral commensal streptococci such as *S. oralis* ATCC 35037 (Fig. 1), strain J22 exhibits stronger inhibition of *Streptococcus mutans* under varying sugar conditions (3, 5). Despite its functional relevance, the whole genome sequence of strain J22 had not been available, limiting mechanistic studies, such as metatranscriptomics.

Strain J22, originally isolated from a human oral cavity (2), was kindly provided by Dr. Jens Kreth (Oregon Health & Science University). The glycerol stock was streaked onto brain heart infusion agar plates supplemented with 5% sheep blood and incubated at 37°C under 5% CO_2_ for 48 h. Genomic DNA was extracted using the MasterPure DNA Purification Kit (Epicenter, USA), beginning with enzymatic lysis using lysozyme and mutanolysin in TE buffer, followed by Proteinase K digestion and RNase A treatment (6). The purified gDNA was subjected to quality assessment using Agilent ScreenTape analysis (Agilent, USA).

The genome of *S. oralis* J22 was sequenced using both PacBio Sequel IIe (Pacific Biosciences, USA) and Illumina NovaSeq X (Illumina, USA) platforms operated at Microgen Inc. (Republic of Korea). For PacBio sequencing, 4 µg of genomic DNA was sheared using Megaruptor 3 (Diagenode), size-selected (7–12 kb fragments) with AMPure PB beads, and prepared with the PacBio SMRTbell prep kit 3.0. The libraries were annealed and sequenced using Sequel II Bind Kit 3.2 and SMRT Cell 8M with 15-h movie time on the PacBio Sequel IIe system. For Illumina sequencing, the TruSeq DNA Nano Kit (Illumina) was used to prepare the libraries, with 100 ng of fragmented DNA, and paired-end sequencing (2 × 150 bp) was performed on the NovaSeq X (Illumina). HiFi reads generated from PacBio were *de novo* assembled using the Microbial Genome Assembly pipeline in SMRT Link v13.0.0.207600, based on the Hierarchical Genome Assembly Process (7). Illumina reads were processed with Trimmomatic v0.38 (ILLUMINACLIP:Adapter.fasta:2:30:10:8:true LEADING:15 TRAILING:15 SLIDINGWINDOW:4:15MINLEN:36) (8). Reads with Phred score ≥30 were retained and used for three rounds of polishing with Pilon v1.22 (9). Circularization was confirmed by identifying overlapping terminal regions, and the genome was rotated to start at the origin of replication based on *dnaA* coordinates.

Genome annotation was performed using Prokka v1.14.6 (10) to predict coding sequences (CDSs), tRNAs, and rRNAs, while InterProScan v5.34-73.0 (11) and PSI-BLAST v2.6.0 (12) were used for further functional annotation, utilizing the EggNOG database v4.5 (13) for orthology.

The *S. oralis* J22 sequence was deposited at GenBank as one circular contig comprised of 1,967,320 bp along with 41.1% GC content, 1,861 CDSs, 61 tRNAs, and 12 rRNAs (Table 1).

**TABLE 1:** Complete genome sequencing, *de novo* assembly, and annotation of *S. oralis* subsp. *tigurinus* strain J22

Category	Feature	*S. oralis* J22
HiFi reads	No. of reads	30,781
	Total bases (bp)	209,722,659
	*N*_50_ (bp)	7,641
Filtered Illumina reads	No. of reads	11,951,272
	Total bases (bp)	1,803,548,598
Assembly	No. of contig	1
Total	Genome size (bp)	1,967,320
	*N*_50_ (bp)	1,967,320
	GC content (%)	41.1
	Mean depth (×)	106.4
	No. of CDs	1,861
	Coding ratio (%)	98.5
	No. of tRNAs	61
	No. of rRNAs	12

## Data Availability

The complete whole-genome sequence of *Streptococcus oralis* subsp. tigurinus strain J22 has been deposited in GenBank under the accession BioProject # PRJNA1094609; GenBank # CP151631; Biosample # SAMN40702978; SRA # SRX27661449 and SRX27661450.
